# Ethics of cancer care: beyond biology and medicine

**DOI:** 10.3332/ecancer.2019.911

**Published:** 2019-03-28

**Authors:** Soumita Ghose, Vivek Radhakrishnan, Sanjay Bhattacharya

**Affiliations:** 1 Administration and Policy, Tata Medical Centre, Kolkata 700156, India; 2 Department of Clinical Haematology and Hematopoietic Cell Therapy, Tata Medical Centre, Kolkata 700156, India; 3 Department of Microbiology, Tata Medical Centre, Kolkata 700156, India

**Keywords:** cancer, ethics, vaccination, screening, global health, LMIC, prevention, India

## Abstract

Treatable cancers are on the rise due to improved early diagnosis and more innovative treatments, and preventative strategies against cancer are becoming a global concern. With the rapidly increasing complexity of cancer treatment, a clear definition of what constitutes ethical cancer care has become a matter of great debate. This situation is more complex in a developing country where healthcare resources are limited. Doctors, nurses and public health professionals engaged in the prevention, screening, diagnosis, treatment and research of cancers are often posed with ethical dilemmas while making complex choices. With a special focus on low- and middle-income countries, this paper is intended to highlight these real-world ethical concerns facing those involved in the management of cancer patients. While taking a neutral view, this paper has adopted a theme-wise approach to discuss barriers in cancer care.

## Introduction

With rapidly increasing incidence rates, high prevalence and delayed diagnosis leading to poor outcomes in developing countries, cancer is a global health challenge of this century. The cost of delivering cancer treatment is estimated to rise globally with a projected total spending of $458 billion by 2030 [1].

With the advent of new technologies and a growing body of public health knowledge, the prevention, screening, diagnosis, treatment and palliation of cancer patients have become increasingly complex. In addition to the burden of disease, in low- and middle-income countries (LMICs), it is often beyond the financial, as well as geographical reach for the majority of population. The rich and poor divide among and between countries has been increasing with the advent of new technologies, cancer drugs and the associated increased cost for the same [1]. Even for the preventable cancers, outcomes vary between developed and developing country populations. The social determinants of the disease go beyond genetic dispositions and are affected by other complex factors such as cultural preferences, lifestyle choices, belief systems, stigma, etc. Furthermore, developing countries lack healthcare infrastructure to support early detection of cancers leading to better treatment outcomes. In the World Innovation Summit for Health 2015, the Qatar Foundation presented that the future of cancer care will be a ‘value challenge’ to the health systems as it will constantly have to address how to provide accessible, equitable and affordable cancer care to populations in need [1].

In this complex socio-economic perspective, establishing the definition of ethical, equitable and acceptable cancer treatment has become a challenge. Therefore, the ethical questions that arise in the care of patients with cancer need to be viewed from multiple contexts.

**Ethics in cancer care**

- Competing interests among global health communities, industries and governments on mandating primary prevention strategies such as tobacco control
- Equitable access for evidence-based screening, diagnosis and treatment of cancer in underprivileged populations
- Personal autonomy and freedom of choice for mandating vaccinations
- Allocating scarce resources among competing needs in low-income settings
- Socio-economic, political and cultural influences on the treatment of cancer

This paper intends to discuss the ethical concerns arising in the prevention, screening, diagnosis and treatment of cancers and in cancer research. With a special focus on LMICs, it is targeted towards all healthcare professionals working in the oncology space; it attempts to address ethical issues faced at multiple levels from health policymakers, government, health professionals, administrators and the global health community at large.

## Tobacco control: challenges from a low middle-income tobacco producing country

**Tobacco Control Fact Sheet: India**

- India currently produces approximately 730 million kg of dry tobacco [2]. It also exports 672 million pieces of bidi (a form of cigarette) and 14,172 tonnes of chewing tobacco in 2010–12 [3, 4]
- This makes India the second largest tobacco producer (730 million kg) and exporter (260 million kg) globally [2]
- As per the 2010–12 data, India produced 110,821 million pieces of cigarettes for domestic production and 9,259 million pieces for exports [3, 4]
- Out of nearly 5.5 million labourers engaged in making bidi, 85% are women and children [3, 4]
- In spite of growing public health concerns, the tobacco industry in India is thriving due to this employment and revenue generating capabilities.

Tobacco is the single most important preventable cause of death globally [5]. Tobacco use is associated with several types of cancers which are very common in LMICs contributing to 50% of all cancers in men and 20% in women [6, 7]. Globally, 90% of lung cancer deaths in men and 80% in women are attributable to smoking [8]. By 2030, tobacco use is estimated to kill around 10 million people a year [6–10]. The epicentre of this tobacco epidemic is LMICs with 70% of estimated deaths and 80% of the 1 billion smokers in the world coming from here [9–13]. Prevention of early tobacco debut and subsequent use of tobacco in youth is one of the critically potential ways of reducing the burden of cancers and other non-communicable diseases in the world. The enactment of the WHO Framework Convention on Tobacco Control (FCTC) treaty in 2003 is the single biggest initiative taken to control the use of tobacco in the world; however, compliance with the WHO FCTC treaty remains bleak and complex in many countries, especially in the developing world. Tobacco control remains a highly sought after trade in countries such as India and China with its potential for generating consistent revenue, to give employment to millions of jobless and the possibility of export to the whole world [14]. Dominated by multinational corporations, the organised sections of the tobacco industry play a huge role supporting its growth. This uniqueness poses a great challenge for public health policymakers and also the governments who constantly struggle to implement legislative controls despite being big advocates for tobacco control. For example, in India, the Cigarettes and Other Tobacco Products Act passed in 2003 was a result of a decade long battle against tobacco, but the Ministry of Labour resisted this act in light of the adverse impact on the labour force of the country [14]. Leaving out tobacco control from the Millennium Development Goals has given the tobacco industry an advantage over the last decade. But with an emphasis on tobacco control in the United Nations’ Sustainable Development Goals, the focus of the global health community is back on the tobacco epidemic [15]. The Sustainable Development Goal 3 targets strengthening the implementation of WHO FCTC in all countries [16]; however, the tobacco industry continues to be a major deterrent for all tobacco control efforts in many countries. Furthermore, not much progress has been done in the UN to address creation of alternative livelihoods for the population employed in tobacco production across the world, including small-scale subsistence farmers [17]. Enormous political will and long-term engagement from a broad range of stakeholders would be required for downsizing the tobacco industry, strict implementation of the law and effectively reducing the use of tobacco. Overall, tobacco control needs to be seen not only as a public health issue but as a fundamental ethical human right.

## Preventive screening and vaccination: economic, socio-cultural and ethical concerns

**Case scenario 1. Health policymaking in the developing world**

In a country like India where the mortality in women with breast cancer is up to 60% over 5 years of diagnosis, most experts believe that screening and early diagnosis would possibly help. Data regarding screening are hugely debated. For men, tobacco related cancers are the largest contributor of morbidity and mortality. Both of these can be targeted by primary and secondary prevention, early diagnosis and treatment strategies. National health programmes in most LMICs have still not included cancer screening programmes as a health policy initiative although governments are looking at various possibilities.

**Ethical dilemma:** In a resource-limited setting, health planners are faced with a dilemma to prioritise funding for preventing high rates of infant mortality through treatment of diarrhoea, better vaccination coverage and preventing other communicable disorders. Maternal mortality and road traffic accidents are several other competing funding priorities of the Govt. Do tax-paying citizens support funding for cancer screening and tobacco cessation at the cost of other programmes?

A growing number of factors have been identified as pre-disposers of cancer through research in genetics, public health and epidemiology. The body of knowledge is constantly increasing on the genetic, lifestyle associated, demographics and epidemiological risk factors important in mediating cancer risks. Today personalised or precision medicine is starting to tailor medical decision making to an individual patient’s risk of disease. However, with these advancements, an increase in ethical questions in the primary and secondary prevention of cancers has also emerged. Many of the cancers are potentially curable if diagnosed and treated at an early stage. This evolving strong evidence for early diagnosis has led to the initiation of cancer screening programmes in many countries. Although these screening programmes are shown to reduce certain cancer incidences and mortality by 50%–60% [18, 19]; it is less cost-effective in LMICs even today, where studies have revealed that population-based screening such as mammography is not cost-effective [20]. As a result, the LMICs are now focusing the screening programmes on specific high-risk populations, hoping to achieve a better early detection rate [20]. This lack of screening and poor awareness in developing countries is often associated with higher cancer-related mortality [21].

Multiple factors emerge as contributors to these low rates of screening and early detection; often these factors are beyond the scope of medicine and deal with complex socio-cultural, economic and political priorities.

### Stigma associated with women’s cancers in the developing world: a deterrent to early detection?

**Stigma—A barrier to cancer screening: Facts from India**

Breast cancer is stigmatised in Indian societies. Treatment of breast cancer often involves the surgical removal of breasts; this compromises the traditional and acceptable image of women in the society [22]. The perceived shame associated with having breast cancer leads to fear of being stigmatised and discriminated [23,24]. Being diagnosed with cancer often leads to social and family rejection, inability to get married and can even lead to divorce [22,25–27]. As a result, Indian women are often very apprehensive to take up voluntary screening services or even discuss concerns related to their breasts or cervix [25–27]. In very traditional Indian cultures, even talking freely about an intimate body part often results in stigma. There are also misbeliefs, myths and religious taboos in certain communities where cancer is seen as a ‘curse’ or a ‘death sentence’ [23–25]. Research on cancer stigma in India points out that patients resist the diagnosis of cancer with the fear that they would be blamed for it [28].

Stigma is increasingly being identified as a threat to health promotion, prevention and control [22, 23]. In many conservative societies, the existing programmes on evidence-based screening suffer from low uptake due to the stigma associated with cancer in women [29, 30]. Sometimes even the perceived fear of cancer treatments such as having a mastectomy, which results in the woman losing one or both of her breasts, leads to a general resistance towards screening [23, 24, 26, 31]. As a result of this resistance, the women present to a doctor at a much advanced stage of the disease and their prognosis is often poor with higher mortality [26, 32]. Also, being physically examined by a male doctor is often culturally unacceptable in many societies which make these women lack the courage and motivation to seek screening services [24, 26]. It is important to understand these complex cultural constructs to be able to design screening and treatment programmes which will ensure better acceptability and uptake.

### Communicating screening results: mitigating over-diagnosis, discrimination and fear

In patients who are asymptomatic, preventive screening tests raise social, psychological and financial concerns. There are complex questions related to communicating positive screening results [33], mitigating stigma and discrimination, motivating people to opt for screening even when there are noted risks of being socially stigmatised, psychologically anxious and financially burdened [25, 26, 34]. Questions such as these are further compounded in more culturally restrictive societies, especially in developing countries, [25, 26, 34] where the common practice does not advocate full disclosure to patients. Results of cancer screening can pose considerable psychological impact on the mind of an otherwise healthy individual. Women with positive cancer screening results have been shown to have significantly elevated levels of anxiety [35]. In a study, women overestimated the risk of dying from breast cancer by 20-fold [36]; in other studies, it was found that women believed a one-time negative result of mammography means they are cancer-free for life [37, 38]. Costs related to psychological issues post-screening are also reported for women with positive PAP smear test results [39]. Furthermore, evidence shows that screening tests may lead to significant harm due to experiencing false positive results, over-diagnosis, costs and deaths related to radiation exposures [40]. Such negative impact may potentially lead to non-adherence to future follow-up on screening and early care seeking behaviour [34]. Thus, it often becomes challenging to ensure that the benefits of cancer screening always outweigh the harms not only at the population level but also at the individual level [34].

### Economic burden of screening and treatment: challenges in a developing world

As cancer burden continues to increase due to the ageing population, lifestyle changes and advanced diagnostic modalities, the economic burden of the disease is on the rise. The developing regions are affected worst with delayed diagnosis leading to low survival rates and compromised quality of life [41]. Ethical issues arise in screening programmes when there is a positive test result which requires further testing and treatment in a population which is non-affording; this situation is often faced in developing countries where preventive cancer screening is not funded by the government and the coverage of privately funded programmes are very limited, and therefore remains a luxury for a majority of the population [42]. In India, an Indian Council of Medical Research (ICMR) report estimates that even with expanded services for cytology for cervical cancer screening, one-fourth of the population cannot be covered once in their lifetime in the near future [43–45]. The healthcare community is constantly torn between the need to conduct cancer screening and the constraints of resources to follow-up on the screen-positive population, as the economic impact of the disease is profound for families. In addition, designing a screening programme also needs to address issues of over-diagnosis and over-treatment which potentially can add more burdens to the existing health system and on the population [46]. Therefore, it is a great challenge for healthcare decision makers to implement a programme which addresses the economic effectiveness and the benefit-harm ratio optimally.

### Vaccination for cancer prevention: invasion in human autonomy?

**Vaccination Fact Sheet: Ethical Considerations in mandating vaccines**

The American Journal of Bioethics discusses the criteria for consideration before mandating a public health intervention. These ethical considerations are often highly debated in case of the HPV vaccination, especially in developing countries [47]

- A clear medical value of the intervention to the individual
- The public health benefit of the intervention
- Consideration of other options to evaluate whether mandate is the only way to achieve compliance and desired results

The first vaccine against the Human Papilloma Viruses (HPV) was approved by the Food and Drug Administration (FDA) in 2006 and was implemented in many countries [48,49]. The global health community, especially in developed countries, had been advocating for mandating it on a mass scale level for the prevention of HPV infections and cervical cancer [50]. There is evidence for population-based screening and vaccination programmes in reducing cervical cancer mortality in both developed and developing countries [18]; however, mandating the use of the HPV vaccine has raised ethical, legal and social dilemmas in healthcare systems [49]. While one section of the healthcare community advocates for its implementation [48]; many have argued that the long-term safety and efficacy of the vaccine are still not completely known, including its period of induced immunity and effects of its co-administration with other vaccines [48, 51, 52]. Social-health advocates have also raised concerns about interfering in individual and parental autonomy by mandating the use of the HPV vaccine in children [48]. In some culturally restricted societies, the vaccine has raised social concerns, inhibiting vaccination of young girls with HPV, as pre-marital sex is socially unacceptable in these communities and parents do not perceive their children as vulnerable to the infection [52, 53]. In addition, there are also significant economic concerns; mainly in developing countries, where such vaccination is not funded by the government as part of mandatory vaccine programmes [48]. Developing countries are the worst affected by cervical cancers with the highest number of deaths, they also lack routine screening due to lack of healthcare funding. Mandating the vaccination in LMICs as a preventive measure has potential to impact a large proportion of the population from getting the HPV infection. However, this has not been achieved in most of the countries due to ethical and economic concerns which are manifold.

**Case Scenario 2. Constraints of treatment funding**

A 21-year-old medical student is diagnosed with B-cell ALL. He developed high-grade fever with elevated blood counts, which on evaluation led to a diagnosis of ALL. After an early initial response, he relapsed while on consolidation chemotherapy. He received salvage chemotherapy and did not achieve a complete response. He is in a good performance status otherwise. The options any financial constraints would be to enter into an appropriate clinical trial or have access to new generation systemic therapies like immunotherapy. Three of the recently approved drugs namely Inotuzumab, Blinatumomab and CAR-Tcells (Kymriah) have shown phenomenal responses in these case scenarios leading to cure, but they all come at a significant financial cost. The other less effective option is salvage conventional chemotherapy. All aforementioned therapies have to be consolidated with a bone marrow transplant which is also an expensive therapy. Our patient comes from a middle-income family which earns $616 per annum and the insurance cover for his family is really not sufficient. There is no universal healthcare covering intensive therapies of cancer in most LMICs. Most of these countries have limited clinical research and clinical trial infrastructure, and if they do, very few have clinical trials involving expensive therapies.

**Ethical dilemma:** The dilemma faced by the treating physician is what to offer this young medical student suffering from a disease, which in conventional terms is fatal, but with adequate financial resources or access can be treated successfully.

**Possible solutions:** Drug and device pricing should have a humane angle considering that most patients in the world cannot afford expensive therapies. Healthcare and device technologies, research methodologies, funding of research, indigenisation of technology, biosimilar drug development, etc. must focus on delivering cost effective therapeutics. Pricing of drugs and medical devices must follow a professional standard of costing instead of only targeting windfall profits. Not adhering to these tenets leads to financially unsustainable treatments for a country forcing them to enforce drug price control regimes.

### Clinical and managerial decision making in cancer treatment: competing priorities

Clinical decision making in cancer is a complex affair, especially in resource-poor settings. With a rapid advancement in the discovery of new cancer drugs with noted clinical benefits, the cost of treatment has also increased significantly [54]. Cancer caregivers are faced with multiple ethical challenges that arise during the course of patient care. A widely practiced model of shared decision making is adopted by many care providers but unavoidable conflict arises when the treatment plan of the physician does not match with the personal choices, economic and social burdens of his patient [55]. To what extent a cancer physician should respect the patient’s right to choose treatment is a very complex judgement. If an adequately informed patient refuses treatment, care providers have merely a limited role to play; this gets more complex for patients who opt for alternate forms of treatment during the course of their illness. LMICs experience 65% of total cancer deaths in the world with 5% of global health resources allocated for cancer control [42, 56]. In 1970, only 15% of new cancer cases were reported from the developing world, this became 56% in 2008 and is predicted to be 70% by 2030 [57–59]. Today, two-thirds of the 7.6 million yearly deaths from cancer happen in LMICs [58–60]. The challenges in the treatment of cancer in developing countries are unique and closing the gap between the rich and the poor is complex. Lack of healthcare infrastructure, low awareness in the general population, uncontrolled exposure to carcinogens, high rates of tobacco use and absence of screening programmes make cancer care in LMICs particularly challenging [42]. Technological advances in oncology are directly associated with increased cost of treatment. The advances often offer enormous benefit to the patients who can afford it; but they also lead to an economic burden on the healthcare systems, the patients’ family and the community as a whole [41]. In countries without universal healthcare coverage, the majority of treatment cost is out of pocket, which lead the family to adverse economic circumstances. The ASEAN Costs In Oncology (ACTION) study, one of the largest observational studies in Asia, which followed household burden of cancer, reports higher financial catastrophes in populations with poorer income, less access to cancer treatment and no insurance coverage [41]. Evidence like this calls for health policy actions from governments focusing on affordable and accessible cancer care. Frequently oncologists have to weigh the burden of treatment with anticipated outcome [61]. One major challenge facing healthcare institutions in developing countries is the enormous number of cancer patients reporting to tertiary care facilities because of lack of infrastructure and expertise at other hospitals to manage these patients. In India, Nearly 0.9 million cancer cases are reported each year [62]; this burden on oncology centres significantly compromises the time spent by physicians with each patient.

Cancer treatments, like many other medical treatments, have seen increase in resource requirements. In societies which are largely not under the umbrella of governmental support or health insurance cover, the financial impact can be debilitating [63, 64]. There is unavoidable conflict when patient’s decisions to forego a viable treatment option due to financial, social and cultural reasons are not respected by the physician.

**Case scenario 3. Ethical dilemma about distributive justice**

In many low- and middle-income countries, cancer patients present in advance stages.

A consultant physician in rural India is faced with prioritising admission for four patients where there are only three in-patient beds available in the hospital. The nearest hospital other than where she practices is more than 100 km. The first patient is a 53-year-old man with lung cancer presenting with severe bone pain which is poorly controlled. He also has vomiting due to morphine and is dehydrated, he has dependent children who are minors and his wife is exasperated. The patient would qualify for hospice care for better symptom management.

**Ethical dilemma:** By the logic of the other three patients having curable and treatable illness, most doctors would like to prioritise other treatment methods over palliation in a developing country. However, the tenet of ‘justice’ as discussed in medical ethics is to be principally interpreted as justice for an individual patient and not necessarily distributive justice of equitable allocation of healthcare resources.

**Possible solutions:** Health systems should be empowered with better evidence based yet socialised medicine which ameliorate symptoms of those that are expected to live, as well as those who are dying.

## Ethical considerations in research on cancer patients

The transition of cancer research from the laboratory into clinical trials, and finally, knowledge translation into clinical practice takes several years if not decades. The statistical chance of a new theory of disease prevention-diagnosis-treatment to succeed is often very low. However, the current body of knowledge is built over an enormous corpus of research endeavours. The ethical conduct of biomedical research has made it mandatory for clinical scientists to reveal the side effects and possible inefficacy of the proposed new intervention to their study subjects. This job is neither easy for the counsellor nor for the counselled. The India-Oxford Cancer Research Network reports ethical challenges as one of the barriers to conducting cancer research in India due to risks of inadequate informed consent, mainly due to lack of awareness and literacy in research subjects [65]. Furthermore, compared to other diseases, the potential to enrol into a drug trial for cancer treatment is much higher; on one hand, this benefits the underprivileged population who otherwise would not have been able to afford the treatment; but on the other hand, an important ethical dilemma is raised when the clinical trial ends and the patient has no means of affording the same drug.

### Research on terminally ill cancer patients

The pathways of caring for the terminally ill is often derived from research done on patients who are healthier than the terminally ill, but there still remain certain aspects of palliative and end-of-life care which call for distinctive observation of palliative patients to answer specific research questions. Due to the high emotional barriers faced by families of terminally ill patients, conducting research on such groups are resisted with taboo, rejection and lack of consent [57]. Palliative care and terminally ill patients are identified as vulnerable, thus demanding greater ethical consideration from fellow researchers and their research perspectives. There are other ethical considerations in research on the terminally ill such as the process of informed consent and who can consent on behalf of a palliative patient, who is often not eligible to consent due to lack of comprehension. The patient/family of a terminally ill patient may also feel obligated towards the researcher which may result in an unwilling or unintended coerced consent. In palliative care, standards of care are more prone to be poor compared to other specialties due to lack of resources being allocated, this might be an ethically troubling issue for palliative care researchers as they find it difficult to alleviate such problems clinically.

## Treatment of palliative and terminally ill cancer patients: a psycho-social turmoil

Many patients with terminal cancer with no possibility of cure or disease control have to be referred to palliative care physicians for alleviation of pain, psychological distress, nutritional derangement, etc. This step necessitates the handover of care from the primary physician and his team to the palliative care team. This transition may be psychologically distressing for cancer patients and their relatives. However, clinical logic and rationalisation of cancer care for a large cohort of cancer sufferers necessitate that this transition be made when necessary. Disclosing these realities to patients and relatives is often difficult but has to be done almost as a daily routine for many. Communicating this prognostic information to patients is an area where ethical concerns often arise. Cancer care providers face dilemma in deciding whether to provide survival statistics, discuss life expectancy and poor prognosis, encourage hope and also to be mindful of various cultural and social barriers. While most studies done on this reveal that many patients want to know about prognostic information and life expectancy; exceptions also exist in the literature where patients did not want a quantitative account of the chances of cure [66]. This is more complex in cases where a patient deteriorates from curative to palliative treatment. There is varied evidence on this in the literature where some studies found that patients preferred their prognosis not to be discussed and a few other studies report patients to be well informed of expected course of illness and aims of treatment [66, 67]. In LMICs with poor literacy levels, an additional confounder worsening the ethical dilemma is the patient or family’s inability to comprehend the prognostic information provided. In addition, economic policies of a healthcare system dictate the finite and often scarce resources to be utilised towards more curative patient groups. This has resulted in a longstanding bias towards allocation of resources to palliative cancer patients. Studies have shown that the hospital expenses of care for a terminally ill cancer patient who died within 24 months were double than those cancer patients who did not die [68], other studies have shown that expenditure for cancer patients under the age of 65 grew exponentially for the terminal year and as death approached [68]. This financial and ethical challenge is faced by care providers while allocating resources to curative cancer patients versus the terminally ill.

## Conclusion

In the World Health Report 2007, three major issues were predicted to be affecting healthcare systems, namely, inequality in delivery of healthcare, the inability of nations to manage the increasing burden of non-communicable diseases, including cancer, and the lack of preparedness of health systems to keep pace with advancements in the field of medical science [69]. The global cancer burden is expected to rise beyond 20 million yearly new cases by 2025 [69]; 70% of all these cases are estimated to be from developing countries, leading to serious challenges affecting the healthcare economy [65]. And yet, many governments have been slow to address the health and economic consequences of cancer and the socio-demographic, political-economic factors that worsen the burden of disease. There is a call for action from governments, policymakers and the global health community to enable healthcare systems to deliver equitable and ethical access to cancer care. In developing countries, there is an imminent need to evaluate more sustainable approaches such as integrating cancer screening and prevention with the primary care infrastructures, which has proven to be effective in many countries [69]. Furthermore, there remains a wide scope of research, especially in the developing country context, on the ethical, socio-cultural, economic and political barriers faced in cancer prevention and screening. There is an overall need to see the disease beyond the scientific endeavours as the disease burden affects not only the individual patients and families but also the healthcare systems, governments, society and economies of these countries.

## Conflicts of interest

The authors do not have any conflict of interest to declare.

## Funding statement

All three authors of this paper are full-time employed by the Tata Medical Centre in the areas of cancer care, infection control, cancer research and healthcare quality control and administration.
